# Avian Influenza Virus Prevalence and Subtype Diversity in Wild Birds in Shanghai, China, 2016–2018

**DOI:** 10.3390/v12091031

**Published:** 2020-09-16

**Authors:** Ling Tang, Wangjun Tang, Xiaofang Li, Chuanxia Hu, Di Wu, Tianhou Wang, Guimei He

**Affiliations:** 1 Laboratory of Wildlife Epidemic Diseases, School of Life Sciences, East China Normal University, No. 3663, North Zhongshan Rd, Shanghai 200063, China; 51181300137@stu.ecnu.edu.cn (L.T.); 51171300141@stu.ecnu.edu.cn (W.T.); 18321525196@163.com (X.L.); antoniahu@163.com (C.H.); 2 Shanghai Wildlife Conservation and Management Center, Shanghai 200072, China; wudifloat@163.com; 3 Institute of Eco-Chongming (IEC), East China Normal University, Shanghai 202162, China

**Keywords:** influenza A virus, surveillance, wild birds, Eastern China

## Abstract

From 2016 to 2018, surveillance of influenza A viruses in wild birds was conducted in Shanghai, located at the East Asian–Australian flyway, China. A total of 5112 samples from 51 species of wild birds were collected from three different wetlands. The total three-year prevalence of influenza A viruses among them was 8.8%, as assessed using real-time polymerase chain reaction (PCR) methods, and the total prevalence was higher in Anseriformes (26.3%) than in the Charadriiformes (2.3%) and the other orders (2.4%) in the Chongmin wetlands. Anseriformes should be the key monitoring group in future surveillance efforts. The peak prevalence of influenza A viruses in Charadriiformes were in April and September, and in other bird orders, the peaks were in November and December. Twelve subtypes of haemagglutinin (HA; H1–H12) and eight subtypes of neuraminidase (NA; N1, N2, N4–N9) were identified in 21 different combinations. The greatest subtype diversity could be found in common teal, suggesting that this species of the bird might play an important role in the ecology and epidemiology of influenza A viruses in Shanghai. These results will increase our understanding of the ecology and epidemiology of influenza A viruses in wild bird hosts in eastern China, and provide references for subsequent surveillance of influenza A virus in wild birds in this area.

## 1. Introduction

Wild birds are generally recognized as the natural reservoir of avian influenza A viruses (AIVs), and most hemagglutinin (HA) and neuraminidase (NA) subtypes have been detected in them [1]. AIVs usually replicate in the epithelial cells of the intestinal tract of wild birds and are excreted in high concentrations in their feces into the water [2]. When other species of waterfowl contact the contaminated water, they might be infected, leading to further viral transmission along transcontinental flyways [3]. The migration routes of waterfowl are usually linked to the geographical spread of a variety of AIV subtypes, including the highly pathogenic H5N1 influenza virus [4]. Therefore, investigation of influenza virus circulation among wild birds on their flyways might help us to understand the mechanisms of the spread of AIVs and provide early warning influenza outbreaks in domestic poultry.

Among birds’ migratory routes, the East Asian–Australian Flyway has the greatest diversity and highest number of migratory birds, involving >40% of the global migratory bird species [5]. Shanghai is a coastal city located under the East Asian–Australasian flyway and is an important stopover and wintering site for migratory birds. According to a survey report, a total of 436 bird species, belonging to 20 orders and 58 families, have been recorded in Shanghai since the early 20th century [6]. Among these birds, more than 160 species of water birds have been recorded in Shanghai, accounting for more than 60% of China’s water birds. Every year, millions of migratory birds and hundreds of species pass through Shanghai. Long-term surveillance of influenza A virus in wild birds in North America, Europe, Australia, and Africa have been conducted [7,8,9], however, current knowledge of the epidemiological surveillance of AIVs in wild birds in eastern China is sparse. With the emergence of influenza A (H7N9) virus in eastern China in early 2013, and then similar H7N9 human isolates being isolated from tree sparrows in Shanghai [10], periodic surveillance of AIVs from wild birds was further strengthened in this region. To address this gap in knowledge, we report the results of surveillance in the last three years (2016 to 2018), which might increase our understanding of the epidemiology of influenza A viruses in wild bird hosts in eastern China, and provide a reference for further surveillance of AIVs in eastern China.

## 2. Materials and Methods

### 2.1. Ethics Statement and Biosafety

Wild birds were captured and sampled with the permission of and approval by the Shanghai Wild Life Conservation and Management Office (2016 [125], 2017 [125], 2018 [125]), and then released after sampling. All experiments were conducted under biosafety level (BSL)-2 conditions.

### 2.2. Sampling Sites

**Figure 1:**
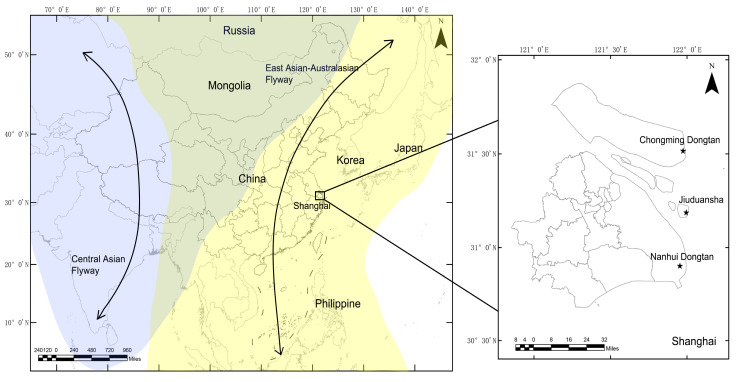
Locations of sampling sites at which the wild birds were monitored in Shanghai, 2016–2018. Shanghai is located in the East Asian–Australasian migratory wild bird flyway, which is marked with a yellow background. The locations of sampling sites are displayed using stars.

The sampling sites are shown in Figure 1. Chongming Dongtan wetland (31°25’–31°38’ N, 121°53’–122°04’ E) is believed to the first important stopover region for certain shorebirds on the northward migration from their non-breeding grounds in Australia or New Zealand [11]. The Jiuduansha wetland (31°06′–31°14′ N, 121°46′–122°15′ E) comprises the three outermost shoals in the Yangtze River estuary and represents an area of about 115 km^2^ [12]. The Nanhui Dongtan wetland (30°51′–31°06′ N, 121°50′–121°51′ E) is located on the northern coast of the Yangtze River. The Jiuduansha and Nanhui Dongtan wetlands are both important stopover sites and wintering habitats for waterfowls. The three wetlands are separated from each other and are all located within the East Asian–Australasian flyway.

### 2.3. Sample Collection

During the routine surveillance of AIV infection of wild birds in Shanghai, China, cloaca and tracheal swab samples were collected from apparently healthy wild birds in Chongming Dongtan, Jiuduansha, and Nanhui Dongtan wetlands (Figure 1). The birds were randomly captured by mist net mainly during their annual migration periods in 2016–2018; for the Charadriiformes, birds were captured during their northward and southward migration periods of late March–April and mid-August–early October annually; for the Anseriformes, birds were captured during the periods of October–December annually; for the other migratory and resident birds, the samples were collected during the two time periods, March–May and August–December. All captured birds were sampled if possible. Due to the randomness of sampling, we could not collect all species present in these sites. The number of birds and their size might contribute to the results, such that small birds might be easier to capture than large ones. The swab samples were immediately placed in tubes containing 2 mL of viral transport media, transported to the laboratory at 4 °C within 24 h, and frozen and stored at −80 °C for further analysis.

### 2.4. Virus Detection

Viral RNAs were isolated from the swab samples on a Magmax-96 Express instrument (Applied Biosystems, Foster City, CA, USA) using the MagMAX™ Pathogen RNA/DNA Kit (Applied Biosystems). Influenza A viruses were detected using quantitative real-time reverse transcription polymerase chain reaction (qRT-PCR) with primers specific for the matrix gene primer and probe set (WHO, 2009) on a 7500 Real-Time PCR instrument (Applied Biosystems). Based on the cycle threshold (Ct) values of matrix genes in these qRT-PCR positive samples, the haemagglutinin (HA) and neuraminidase (NA) subtypes of some isolates were determined by sequencing. Briefly, the selected positive viral RNAs of Influenza A viruses were first transcribed into cDNA using the Uni12 primer (5’-AGC AAA AGC AGG-3’) and a PrimeScript™ II 1st Strand cDNA Synthesis Kit (Takara, Shiga, Japan), and then the subtypes were determined using specific primers for HA and NA [13,14]. Each PCR reaction contained 1 μL of cDNA, 1 μL each of forward and reverse primers, 12.5 μL of Taq HS Perfect Mix (Takara), and 10.5 μL of Rnase-free water in a final volume of 25 μL. PCR products were sequenced using a BigDye termination kit (Applied Biosystems) on an ABI 3730 sequence analyser (Applied Biosystems, Foster City, CA, USA). For the H5 and H7 subtypes, the cleavage sites of HA were further sequenced to screen for potential highly pathogenic avian influenza (HPAI) viruses.

### 2.5. Sequence Analysis

The sequence data were aligned and analysed using the DNAMAN program (version 6.0), and then compared with the sequences in GeneBank (https://www.ncbi.nlm.nih.gov/) and the Global Initiative on Sharing Avian Influenza Data (GISAID) EpiFlu database (https://platform.gisaid.org/epi3/frontend#2de575) using the BLAST algorithm to identify their subtypes.

### 2.6. Statistical Analysis

The prevalence of influenza A viruses in these wild bird samples was estimated from the ratio of positive to the total number of samples, with the exact binomial confidence intervals of 95%. Friedman and Chi-square tests were used to compare the prevalence among different sampling sites and bird orders. Corresponding analysis was used to assess the association between bird orders and the virus subtype in a two-dimensional plot, and the subtype diversity among different wild bird species was evaluated by the Shannon entropy. All statistics were carried out in SPSS software (version 23.0, SPSS Inc., Chicago, IL, USA). For all analyses, *p* < 0.05 was considered significant. Graphs were produced using SigmaPlot 12.0 software (Systat Software, Inc., San Jose, CA, USA). The Geographical maps were plotted using ArcGIS 10.2 software (ESRI, Aylesbury, UK).

## 3. Results and Discussion

### 3.1. Sampling Overview

From March 2016 to December 2018, a total of 5112 wild bird samples belonging to 51 species of 10 orders were collected. Among these samples, most were collected from Anseriformes (47.7%), of which the majority were common teal (*Anas crecca*) (76.0%), and from Charadriiformes (30.6%), of which 59.5% were the great knot (*Calidris tenuirostris*). Fewer samples were collected from the other bird orders (21.8%), such as Ciconiiformes, Gruiformes, Podicipediformes, Strigiformes, and Passeriformes.

### 3.2. Prevalence Overview

The overall three-year prevalence of influenza A viruses in these wild bird samples was 8.8% as assessed using qRT-PCR (Table 1), and the annual influenza A virus prevalence was 7.5% (122/1622), 9.4% (155/1649), and 9.5% (175/1841) in 2016, 2017, and 2018, respectively. The total prevalence in this study was apparently higher than previous reports in South-eastern China and Central China located on the East Asian–Australasian flyway, which were about 0.65–8% [15,16,17]. The difference in prevalence might be explained by the different sampling species, diagnosis methods, and type of samples. For example, the qRT-PCR method used in this study is highly sensitive and specific for the analysis of bird samples, as described previously [18]; and more positive samples might be detected from swab samples than faecal samples due to their difference in freshness. Furthermore, the most important effect of the difference in the total prevalence should be attributed to the different sampling species. The highest prevalence in the studies mentioned above was detected in the Bean Geese, which usually breed in northern Europe and Asia. However, the dabbling ducks in this study mainly breed in northeast China. It is possible that the ancestor of the virus might be different in different breeding grounds, additional studies would be needed to explain these differences in prevalence among different species.

The prevalences among different orders in the Chongming Wetland and different sampling sites in the Anseriformes were compared by Chi-square tests, and the results showed that these differences were statistically significant (*p* < 0.05). In the Chongmin wetlands, the total prevalence was higher in Anseriformes 26.3% (30/114) than in the Charadriiformes 2.3% (36/1564) and the other orders 2.4% (27/1112). The results further confirmed that the high positive rate of influenza A virus was related to the order of wild birds, and the Anseriformes should play a major role in maintaining avian influenza viruses in this area and would be the key monitoring group in future surveillance efforts. For the Anseriformes, the Chongming Wetland (26.3%, 30/114) was greater than that in the Jiuduansha (17.6%, 152/862) and Nanhui Dongtan Wetlands (14.2%, 207/1460) (Table 1). The highest prevalence was found in the Chongmin wetland, which might be result of the emergence of a new H7 subtype virus (8 strains) and the sampling size. In future surveillance, more samples should be collected to avoid sampling bias in this region.

### 3.3. Seasonal Variation

**Figure 2:**
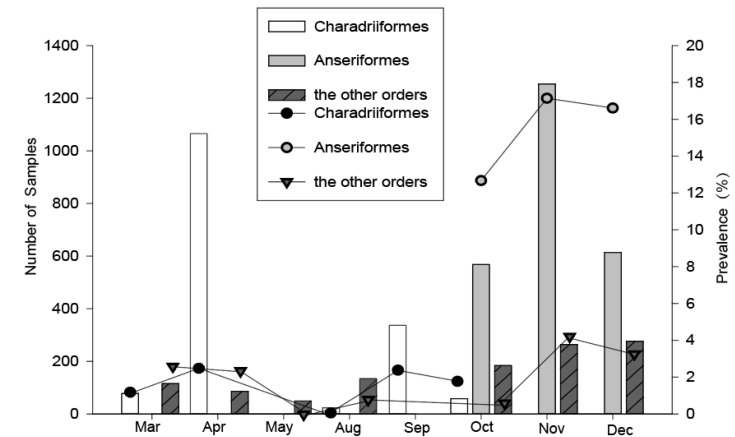
Temporal variation of the overall number sampled and the avian influenza A virus (AIV) prevalence among wild birds during 2016–2018 in Shanghai. Data from 2016–2018 were pooled, the AIV positive rates were detected using quantitative real-time reverse transcription polymerase chain reaction (qRT-PCR).

For the Charadriiformes, birds were captured during their northward and southward migration periods annually. However, about three times as many birds were caught in the northward migration period in spring compared with that in the southward migration period in fall (Figure 2), because the total number of shorebirds in the southward migration was less than that in the northward migration in the Chongming wetland, and a study has shown that this area is less important as a stopover site for shorebirds during their southward migration [11]. The highest prevalence of influenza in Charadriiformes was found in April (2.4%) and September (2.4%), and the lowest in August (0%). For the Anseriformes, we captured the birds during the periods of October–December, and the prevalence of influenza A virus in November (17.2%) and December (16.6%) was higher than that in October (12.7%). For the other migratory and resident birds, we collected the samples during the two time periods, March–May and August–December, and the highest prevalence of influenza A virus could be found in November (4.2%) and December (3.3%). Therefore, the timing of the influenza A virus peak prevalence in Charadriiformes were in April and September, but in other bird orders were in November and December.

### 3.4. Subtype Diversity

**Figure 3:**
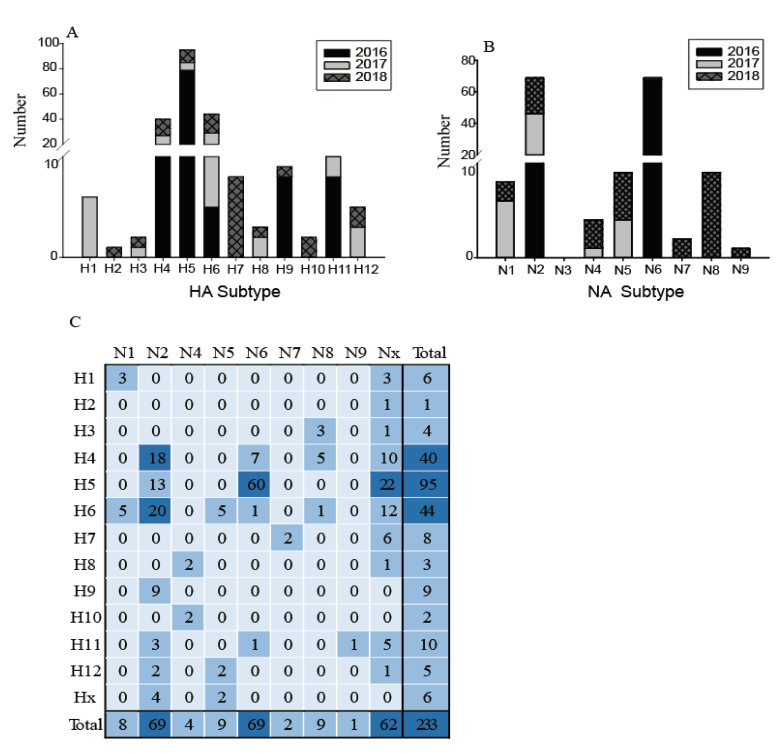
Distribution of haemagglutinin (HA) subtypes, neuraminidase (NA) subtypes and HA/NA subtype combinations during influenza A virus surveillance in wild birds in Shanghai, 2016–2018. (**A**) Number of HA subtypes; (**B**) number of NA subtypes; (**C**). Number of HA/NA subtype combinations. HA/NA subtype combinations (blue) were colored according to the frequencies of detection.

The low quantities of viral RNA of some samples, only 233 of these testing positive for influenza A virus were selected for further influenza virus subtyping. Twelve subtypes of HA (H1–H12) and eight subtypes of NA (N1, N2, N4–N9) were identified in 21 different combinations (Figure 3). The most common subtypes of HA were H4, H5, and H6, followed by H11, H7, and H9 (Figure 3A). Meanwhile, for the NA subtypes, N2 and N6 were the most common, followed by N1, N5, and N8 (Figure 3B). H4, H5, H6, and N2 subtypes were identified in each year, indicating that wild birds might be a reservoir for these common subtype viruses. The subtype distribution in this study was similar to that found in the Eurasian region and in North America [7,8], such that H4 and H6 are abundant globally. In contrast, the H1, H2, H7, H10, and N7–N9 subtypes were only found in particular years. The H13–H16 and N3 subtypes were not found in this surveillance. Figure 3 also shows an increasing annual trend in HA and NA subtype diversity, such as H1, H3, H8, H12, and N1, N4, and N5 subtypes being identified since 2017; while H2, H7, H10, and N7, N8, and N9 were only identified in 2018. This increasing diversity of subtypes might provide the potential for reassortment of these viruses.

The most common subtype combinations were H5N6, H4N2, and H6N2 (Figure 3C), which together accounted for 42.1% of all the isolated viruses. H4N2 and H6N2 viruses were found during the entire study period; however, the H5N6 highly pathogenic avian influenza (HPAI) viruses were detected only in 2016 (for detailed information, please refer to our previous study [19]). Except for these H5N6 viruses, all other H5 and H7 subtypes were characterized as low pathogenicity avian influenza (LPAI), indicating that the HPAI viruses are not common in the wild birds in this region; however, sporadic infections in some years still deserve special attention. During 2013–2014, the HPAI subtype H5N8 viruses were detected from migratory ducks in Shanghai, which might be the earliest detection of HPAI H5N8 virus in wild birds in East Asia [20]. Therefore, more intensive surveillance should be carried out in wild birds and it is necessary to monitor the evolution of H7 and H5 AIVs, focusing on their potential pathogenicity to mammals.

### 3.5. Species Differences

**Figure 4:**
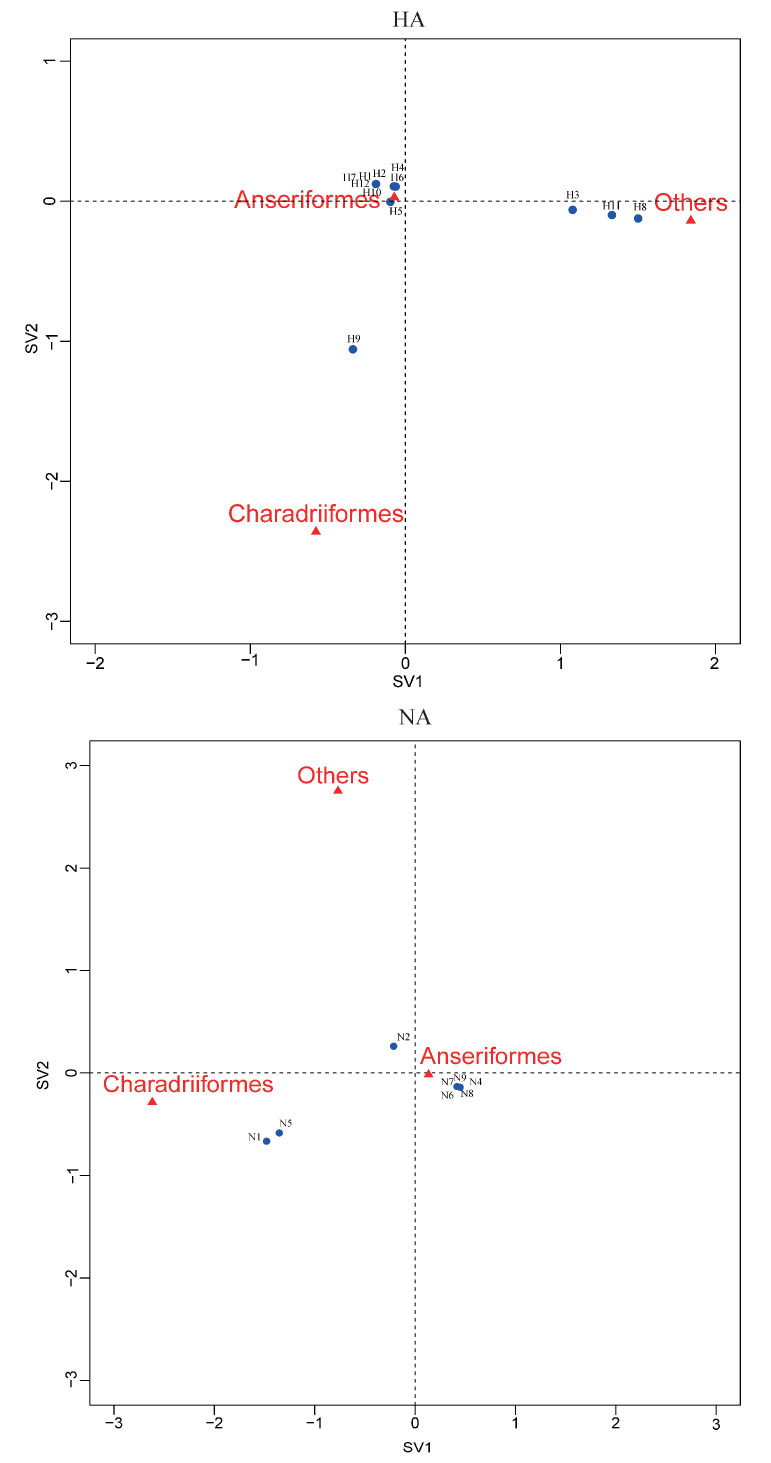
Correspondence plot showing the association between bird orders and HA, NA subtypes in two dimensions (singular value (SV)1 and SV2). All subtypes were detected during influenza A virus surveillance in wild birds in Shanghai, 2016-2018.

Correspondence analysis of HA, NA subtypes and bird species indicated an association of H9, N1, and N5 viruses with Charadriiformes, and H3, H11 and H8 viruses with other orders (Figure 4). The other HA and NA subtypes might be associated with Anseriformes, which might support that Anseriformes were the natural reservoir sensitive to infection with most AI virus strains. Furthermore, we also found that they all fell around the centre of the correspondence plot, perhaps indicating there was no clear host–virus subtype associations among them. To confirm the results, more epidemiological data from the surveillance of wild birds in this region are needed.

At the bird species level, we found that AIV infection was found in 22 species (Table 2). AIV positive samples could be found each year in common teal (*Anas crecca*), spot-billed duck (*Anas poecilorhyncha*), mallard (*Anas platyrhynchos*), eurasian wigeon (*Anas penelope*), and northern shoveler (*Anas clypeata*), and the prevalence rates were relatively high (14.7–26.3%). For the great knot (*Calidris tenuirostris*), dunlin (*Calidris alpina*), and red knot (*Calidris canutus*), although AIV positive samples were found annually in these species and they are also the more common migratory species in the Congming Dongtan wetland, their prevalence of AIV infection was relatively low (1.4% to 3.2%). The shorebirds samples in the present study were collected mainly during their northward migration from Australia or New Zealand, and the overall prevalence of AIV in shorebirds in Australia is also relatively low, at approximately 0.6 ± 0.1% [21].

As shown in Table 2, 12 HA (H1–H12) and 8 NA (N1, N2, N4–N9) could be identified in the samples from the common teal. To explore the subtype diversity among different wild bird species, the diversity index (Shannon entropy values) were analyzed. The results showed that the Shannon entropy values in common teal were higher than the other bird species, indicating the greatest subtype diversity in common teal. The common teal (*Anas crecca*) is one of the most abundant migratory duck species in Shanghai, accounting for 48.6% of the total individuals recorded in the Jiuduansha wetland [22], which were caught in substantial numbers among the waterfowl population in Shanghai in this study. Moreover, common teal has been shown play a central role in the spread of influenza A virus in nature [23], and its prevalence of influenza A virus in this study was also high (15.3%); therefore, this species might play an important role in the ecology and epidemiology of influenza A viruses in Shanghai and should be regarded as the key surveillance duck species in the future, which could help to understand AIV epidemiology in one of the major reservoir hosts.

Spot-billed duck (*Anas poecilorhyncha*) and mallard (*Anas platyrhynchos*) are also common migratory species in Shanghai. Although the prevalence rates of AIV in the two kinds of ducks were higher than that in common teal, less diversity of HA and NA were found in these species. The possible reason is that fewer spot-billed duck and mallard were captured. This type of sampling bias should be ruled out in future epidemiological surveys.

Furthermore, for some representative subtypes detected in this study, genetic analyses have been performed in our previous published manuscripts, such as H5N6 viruses [19], H8N4 virus [24], H6 subtypes [25], H7 and H8–H12 subtypes (data not shown). From these analyses, we found that most of these strains were all clustered in the Eurasian lineage, but there were also a few gene segments belonging to the North American lineage. Most strains circulating in Shanghai were closely related to the viruses in poultry and wild birds from Korea, Japan, Mongolia, Bangladesh, and central China located in the East Asian–Australian flyway. All these results further confirmed that migratory birds should play a very important role in the spread of avian influenza virus along migratory flyways, even in transcontinental transmission.

In summary, the present study demonstrated the epidemiology of influenza A viruses in wild bird hosts in Shanghai. Long-term surveillance of influenza A virus in wild birds is required to better understand the ecology and epidemiology of AIV in Eastern China, and will also contribute to the development of successful control measures and strategies to manage and reduce the impact of (HPAI) virus outbreaks.

## Figures and Tables

**Table 1:** Prevalence of influenza A virus in wild birds in Shanghai, 2016–2018.

Sampling Sites ^†^	Orders	2016			2017			2018			Total		
		No. of Samples	No. of Positive	Prevalence (95% CI, %)	No. of Samples	No. of Positive	Prevalence (95% CI, %)	No. of Samples	No. of Positive	Prevalence (95% CI, %)	No. of Samples	No. of Positive	Prevalence (95% CI, %)
CM	Anseriformes ^‡^							114	30	26.3 (17.8–34.8)	114	30	26.3 (17.8–34.8)
	Charadriiformes	336	7	2.1 (0.4–3.8)	519	12	2.3 (0.9–3.7)	709	17	2.4 (1.2–3.6)	1564	36	2.3 (2.1–2.4)
	Other orders	584	10	1.7 (0.6–2.9)	460	17	3.7 (1.9–5.5)	68	0	0.0 (0.0–5.3)	1112	27	2.4 (0.0–3.9)
	Total	920	17	1.8 (1.6–2.2)	979	29	3.0 (1.9–4.1)	891	47	5.3 (0.0–26.0)	2790	93	3.3 (1.4–5.4)
JDS	Anseriformes	219	45	20.5 (15.0–26.1)	240	42	17.5 (12.5–22.5)	403	65	16.1 (12.4–19.8)	862	152	17.6 (15.5–20.6)
NH	Anseriformes	483	60	12.4 (9.4–15.5)	430	84	19.5 (15.7–23.4)	547	63	11.5 (8.8–14.3)	1460	207	14.2 (9.5–19.4)
Total		1622	122	7.5 (1.0–22.2)	1649	155	9.4 (3.1–23.5)	1841	175	9.5 (4.8–17.1)	5112	452	8.8 (7.5–10.1)

^†^ JDS refers to Jiuduansha wetland, CM refers to Chongming Dongtan wetland and NH refers to Nanhui Dongtan wetland. ^‡^ Anseriformes’ samples from Chongming wetland were collected just in December in 2018.

**Table 2:** Subtypes of influenza A viruses in wild birds in Shanghai, 2016–2018.

Orders.	Common Name	Scientific Name	No. Samples Collected in 2016–2018	No. AIV Positive	Prevalence (95% CI, %)	HA Subtypes (n)	NA Subtypes (n)	Shannon Entropy Values of the Subtype Diversity (HA, NA)
Anseriformes	Common teal	*Anas crecca*	1852	283	15.3 (13.6–16.9)	H1 (6), H2 (1), H3(1), H4 (25), H5 (71), H6 (29), H7 (8), H8 (2), H9 (3), H10 (1), H11 (6), H12 (3)	N1 (6), N2 (35), N4 (3), N5 (5), N6 (57), N7(2), N8 (8), N9 (1)	0.73, 0.60
	Spot-billed duck	*Anas poecilorhyncha*	160	42	26.3 (19.1–33.4)	H4 (13), H5 (9), H6 (7)	N2 (10), N5 (1)	0.46, 0.13
	Northern pintail	*Anas acuta*	50	5	10.0 (0.4–19.3)	H3 (1), H9 (1)	N2 (2)	0.30, 0
	Mallard	*Anas platyrhynchos*	62	15	24.2 (12.7–35.7)	H3 (1), H5 (2), H10 (1), H12 (2)	N4 (1), N6 (3)	0.58, 0.24
	Eurasian wigeon	*Anas penelope*	74	9	12.2 (4.0–20.3)	H5 (4), H6 (1), H11(1)	N2 (3), N6 (3)	0.38, 0.30
	Northern shoveler	*Anas clypeata*	116	17	14.7 (7.8–21.5)	H4 (1), H5 (4), H6 (4), H9 (2)	N2 (5), N6 (5)	0.55, 0.30
	Common pochard	*Aythya ferina*	28	4	14.3 (0.0–29.0)	H5 (1)	N2 (1)	0, 0
	Falcated teal	*Anas falcata*	34	6	17.7 (3.4–31.9)	H6 (1), H9 (2)	N2(2)	0.28, 0
	Gadwall	*Anas strepera*	26	2	7.7 (0.0–19.9)			
	Mandarin	*Aix galericulata*	32	6	18.8 (3.7–33.8)	H5 (1), H6 (1)	N2 (4), N5 (1), N6 (1)	0.30, 0.38
	Tufted duck	*Aythya fuligula*	2					
Charadriiformes	Great knot	*Calidris tenuirostris*	930	22	2.4 (1.3–3.4)		N1 (2), N2 (2), N5 (2)	0, 0.48
	Red knot	*Calidris canutus*	124	4	3.2 (0.0–6.7)		N2 (1)	-, 0
	Dunlin	*Calidris alpina*	114	1	0.9 (0.0–3.0)	H5 (1)	-	0, -
	Terek sandpiper	*Xenus cinereus*	86	1	1.2 (0.0–4.0)	H9 (1)	N2 (1)	0.55, -
	Sharp-tailed Sandpiper	*Calidris acuminata*	22	1	4.6 (0.0–15.5)			
	Bar-tailed godwit	*Limosa lapponica*	80	1	1.3 (0.0–4.3)			
	Ruddy turnstone	*Arenaria interpres*	24	2	8.3 (0.0–21.5)			
	Whimbrel	*Numenius phaeopus*	26	4	15.4 (0.0–31.2)			
	Common snipe	*Gallinago gallinago*	22					
	Red-necked stint	*Calidris ruficollis*	30					
	Greenshank	*Tringa nebularia*	24					
	Sanderling	*Calidris alba*	2					
	Wood sandpiper	*Tringa glareola*	18					
	Golden plover	*Pluvialis dominica*	14					
	Mongolian plover	*Charadrius mongolus*	6					
	Calidris Ferruginea	*Calidris ferruginea*	4					
	Grey plover	*Pluvialis squatarola*	20					
	Crake	*Metopidius indicus*	18					
Gruiformes	Peale	*Fulica atra*	106	14	13.2 (6.3–20.1)	H3 (1), H5 (2), H8 (1), H11 (3)	N2 (3)	
	Common moorhen	*Gallinula chloropus*	82	7	8.5 (1.9–15.2)	H6 (1)		
Galliformes	Pheasant	*Phasianus colchicus*	2					
Ciconiiformes	Spotted redshank	*Tringa erythropus*	12					
	Little egret	*Egretta*	110	5	4.6 (0.2–8.9)	H4 (1)	N8 (1)	
	Green-backed heron	*Butorides striatus*	16					
	Night heron	*Nycticorax nycticorax*	14					
Podicipediformes	Little grebe	*Tachybaptus ruficollis*	2					
Strigiformes	Oriental scops owl	*Otus sunia*	2					
Columbiformes	Bead neck dove	*Streptopelia chinensis*	118					
	Oriental turtle-dove	*Streptopelia orientalis*	2					
Upupiformes	Eurasian hoopoe	*Upupa epops*	2					
Passeriformes	Yellow wagtai	*Motacilla flava*	244					
	Gray wagtail	*Motacilla cinerea*	30					
	White wagtail	*Motacilla alba*	4					
	Chinese bulbul	*Pycnonotus sinensis*	90	1	1.1 (0.0–3.8)			
	Tree sparrow	*Passer montanus*	148					
	Blackbird	*Turdus merula sowerbyi*	92					
	Grey-backed thrush	*Turdus hortulorum*	12					
	Black-tailed hawfinch	*Eophona migratoria*	12					
	Crested myna	*Acridotheres cristatellus*	6					
	Long-tailed shrike	*Lanius schach*	6					
Total			5112	452	8.8 (8.1–9.6)			
